# Knowledge Tensor-Aided Breast Ultrasound Image Assistant Inference Framework

**DOI:** 10.3390/healthcare11142014

**Published:** 2023-07-13

**Authors:** Guanghui Li, Lingli Xiao, Guanying Wang, Ying Liu, Longzhong Liu, Qinghua Huang

**Affiliations:** 1 School of Computer Science, Northwestern Polytechnical University, Xi’an 710129, China; guanghui.li@mail.nwpu.edu.cn; 2 School of Artificial Intelligence, OPtics and ElectroNics (iOPEN), Northwestern Polytechnical University, Xi’an 710072, China; 3 Department of Ultrasound, The Eighth Affiliated Hospital, Sun Yat-sen University, Shenzhen 518033, China; lingli_xiao22@163.com (L.X.); liuyzdby@163.com (Y.L.); 4 Department of Ultrasound, Sun Yat-sen University Cancer Center, State Key Laboratory of Oncology in South China, Collaborative Innovation Center for Cancer Medicine, Guangzhou 510060, China; wanggy2@sysucc.org.cn

**Keywords:** knowledge tensor, BI-RADS, generalization inference

## Abstract

Breast cancer is one of the most prevalent cancers in women nowadays, and medical intervention at an early stage of cancer can significantly improve the prognosis of patients. Breast ultrasound (BUS) is a widely used tool for the early screening of breast cancer in primary care hospitals but it relies heavily on the ability and experience of physicians. Accordingly, we propose a knowledge tensor-based Breast Imaging Reporting and Data System (BI-RADS)-score-assisted generalized inference model, which uses the BI-RADS score of senior physicians as the gold standard to construct a knowledge tensor model to infer the benignity and malignancy of breast tumors and axes the diagnostic results against those of junior physicians to provide an aid for breast ultrasound diagnosis. The experimental results showed that the diagnostic AUC of the knowledge tensor constructed using the BI-RADS characteristics labeled by senior radiologists achieved 0.983 (95% confidential interval (CI) = 0.975–0.992) for benign and malignant breast cancer, while the diagnostic performance of the knowledge tensor constructed using the BI-RADS characteristics labeled by junior radiologists was only 0.849 (95% CI = 0.823–0.876). With the knowledge tensor fusion, the AUC is improved to 0.887 (95% CI = 0.864–0.909). Therefore, our proposed knowledge tensor can effectively help reduce the misclassification of BI-RADS characteristics by senior radiologists and, thus, improve the diagnostic performance of breast-ultrasound-assisted diagnosis.

## 1. Introduction

The incidence of breast cancer is already at the top of the list among women. According to the International Agency for Research on Cancer (IARC), the age-standardized incidence of breast cancer reached 5.2% in 2020 and will continue to grow. Comparatively, breast cancer has a high early-cure rate among all cancers [1]. According to the American Cancer Society (ACS), the five-year relative survival rate of patients with early-stage breast tumors is about 99% after treatment. Once breast cancer progresses to the distant stage, however, the five-year relative survival rate of patients drops to 30% [2], and the pain caused by the lesion itself and medical interventions can have serious negative effects on the patient’s body and mind. Therefore, early detection and treatment of breast cancer are essential to improve the survival rate and quality of life of patients and help to increase the life expectancy of women.

In breast cancer screening, computed tomography (CT), magnetic resonance imaging (MRI), and ultrasound (US) are common medical imaging tools [3]. Among them, CT and MRI have high imaging resolution. Still, they are costly and complicated to operate, and the former has radiation, so they are more often used in the diagnosis of middle- and late-stage breast cancer. In contrast, US is low-cost, radiation-free, simple-to-operate, and has a higher diagnostic accuracy for breast ultrasound (BUS) imaging, so BUS is widely used in the screening and initial diagnosis of breast tumors in the early stages [4]. In addition, BUS imaging is also commonly used in postoperative follow-up to follow up on the results of breast tumor surgery [5]. Since BUS images are obtained as reconstructed images from ultrasound signals, radiologists need to interpret the image features based on their knowledge and experience in the field to obtain medical imaging reports of the lesion and give diagnostic opinions. However, the same problems that exist in other US imaging-assisted diagnostics exist in BUS-assisted diagnostics, i.e., the imaging quality of the US image has a decisive impact on the performance of the assisted diagnosis and is highly dependent on the practice experience and scanning technique of the physician operating the ultrasound probe [6]. In other words, a senior radiologist performing a BUS scan is likely to obtain higher-quality images and arrive at a more accurate medical diagnosis than a junior radiologist.

In the field of medicine, to address the influence of subjective factors such as experience and, thus, reduce misdiagnosis and underdiagnosis, doctors try their best to summarize their experiences and form diagnostic protocols to help young doctors improve their abilities as soon as possible. The Breast Imaging Reporting and Data System (BI-RADS) [7], published by the American College of Radiology (ACR), has become one of the main standards for the diagnosis of BUS, and the BI-RADS uses different scores to describe the presentation and significance of a particular feature of a BUS image, thus creating a scoring system that allows the imaging physician to make a comprehensive judgment of the different features of BUS and give a more accurate and objective diagnosis. BI-RADS takes full account of medical experience and knowledge and has strong interpretability, but identifying BI-RADS characteristics is still not an easy task. In addition, the operator’s technique, imaging quality, and lesion characteristics on ultrasound also have a non-negligible impact on the auxiliary diagnosis of BUS images [8,9].

The advent of computer-aided diagnosis (CAD) has greatly eased the workload of radiologists and improved diagnostic performance [10,11]. With the rise of artificial intelligence in recent years, CAD has also introduced deep models to further improve the performance of BUS-assisted diagnosis [12,13,14,15]. However, deep models currently still have certain limitations, such as the need for a large number of high-quality datasets to train models with good performance, poor resistance to noise interference, and end-to-end characteristics that make them uninterpretable [16]. Combining CAD with BI-RADS is a viable approach. Usually, we need high-quality annotation to train high-performance BI-RADS characteristic recognition models [17]. However, obtaining high-quality annotations is usually a difficult task. One possible approach is to train a high-performance BI-RADS characteristic recognition model from existing deep models [18,19,20], but this still requires a significant initial investment in annotating [21,22]. Moreover, even with valuable medical semantic characteristic data, using them for assisted diagnosis is a difficult problem. Due to the problems of fewer dimensions and fewer data, traditional machine learning models, such as k-nearest neighbors (KNNs) [23] and support vector machine (SVM) [24], as well as deep learning models, are usually unable to effectively mine the information contained in them and are instead very prone to overfitting. The training samples for KNN are vectors in a multidimensional feature space, each with a class label. The training phase of the algorithm consists of storing only the feature vectors and class labels of the training samples. In the classification phase, k is a predefined constant, and the classification of an unlabeled vector is achieved by assigning the most frequent label of the k training samples closest to that query point. Generally, the classification accuracy of KNN can be significantly improved if the distance metric is learned with specialized algorithms, such as large marginal nearest neighbor or neighborhood component analysis. One drawback of the basic “majority voting” classification method occurs when the distribution of classes is skewed. That is, examples of a more frequent class tend to dominate the prediction of new examples because they are common among the k nearest neighbors due to their large number. The idea of SVM, on the other hand, is to map the data into a high-dimensional space and separate the data by constructing a hyperplane in the high-dimensional space to achieve efficient classification. Usually, the computation in the high-dimensional space is very large, while SVM uses kernel functions to cleverly avoid directly computing the high-dimensional space tensor to achieve nonlinear classification. This allows the algorithm to fit the maximum interval hyperplane in the transformed feature space. The transformation can be nonlinear, while the transformation space is high-dimensional; although the classifier is a hyperplane in the transformed feature space, it can be nonlinear in the original input space. It is worth noting that the higher-dimensional feature space increases the generalization error of the support vector machine, but the algorithm can still perform well given a sufficient number of samples. So, it is important to fully exploit the empirical knowledge of senior radiologists to improve the accuracy of BI-RADS-based diagnoses from junior radiologists.

In this paper, we develop a novel knowledge tensor-aided breast ultrasound image assistant inference framework. We first construct the knowledge graphs [25] with BI-RADS characteristics annotated by senior and junior radiologists, respectively. Then, we map knowledge entities and relations to embedding spaces [26]. TuckER [27] decomposition is employed to reconstruct knowledge entities and relations to tensors. At the same time, the diagnosis of target entities based on spatial distance could be inferred. Experiments show that the diagnostic performance of the knowledge tensor is significantly higher for senior radiologists than for junior radiologists. By correcting the model with knowledge tensor scores, the corrected knowledge tensor diagnostic performance of junior radiologists is significantly better than the original one. In conclusion, the framework proposed in this paper can significantly improve the performance of BI-RADS score breast tumor assistant diagnosis for junior radiologists, which is important for clinical practice and could improve the generalization performance of the framework.

## 2. Materials and Methods

### 2.1. Data Collection

Our data are collected from BUS reports of patients at the Sun Yat-sen University Cancer Center between February 2014 and March 2017. The BUS scans were performed with a LOGIQ E9 scanner (GE, Boston, MA, USA) or iU22 xMATRIX scanner (Philips, Amsterdam, Netherlands). All machines are equipped with a high-frequency (6–14 MHz) linear array transducer. Given this range of transducer frequency, the axial resolution is approximately 0.165–0.385 mm. All scans were performed by one of two senior radiologists (Longzhong Liu, with 25 years of experience in US diagnosis, and Ying Liu, with 10 years of experience in US diagnosis). Further, each scan was re-labeled by one of two junior radiologists (Lingli Xiao, with three years of experience in US diagnosis, and Guanying Wang, with two years of experience in US diagnosis) independently.

### 2.2. Statistical Analysis

The statistical analysis is conducted with SPSS 27.0 [28]. Delong’s test [29] with binomial exact confidence intervals is utilized to compare the area under the curve (AUC) of the receiver operating characteristic curve (ROC) of different knowledge tensor-aided inference models.

### 2.3. Knowledge Tensor-Aided Breast Ultrasound Image Assistant Inference Framework

**Figure 1:**
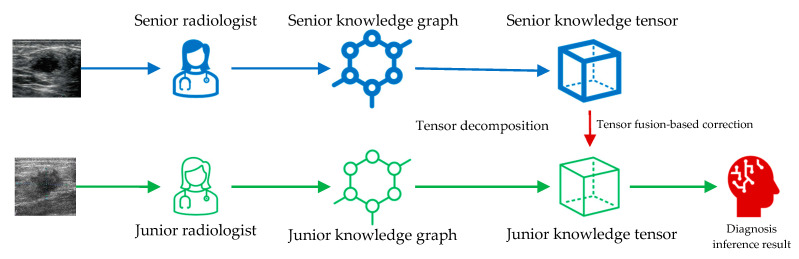
The knowledge tensor-aided BUS image assistant inference framework. BUS images are annotated by senior and junior radiologists to construct medical knowledge graphs. Then, independent knowledge tensors are built to infer the diagnosis results. The senior knowledge tensor is utilized to correct the junior knowledge tensor through tensor fusion. The diagnosis inference results are finally generated.

The framework we proposed relies on knowledge tensor inference for medical-image-assisted diagnosis. Knowledge tensor is a knowledge graph inference method based on tensor decomposition. The construction and inference methods of knowledge tensor are elaborated in Section 2.3 and Section 2.4. The entire framework is shown in Figure 1. Knowledge from senior radiologists and junior radiologists is collected and utilized to construct knowledge graphs. That is to say, the senior annotations are utilized to construct the senior knowledge graph, and the junior annotations are utilized to construct the junior knowledge graph. The two knowledge graphs were independent when we constructed them. Then, tensor decomposition is utilized to decompose each piece of knowledge into tensors. Senior knowledge tensors are next used to correct the tensor space mapping of junior knowledge tensors. Finally, corrected junior knowledge tensors could infer the diagnosis results.

### 2.4. Construction of the Knowledge Graph

**Figure 2:**
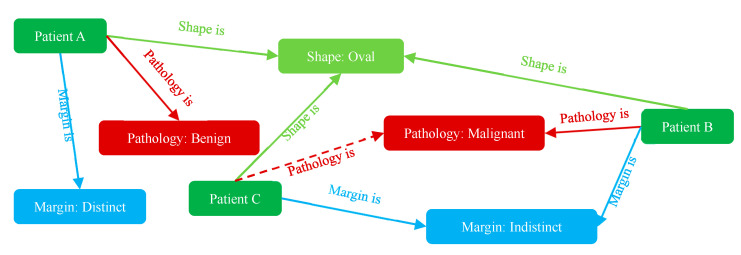
A simple knowledge graph representing BUS image reports. Two BI-RADS characteristics are employed to represent the image, and the benign and malignant entities are employed to describe the pathology results of each lesion. Solid arrows indicate known determined relationships, and dashed arrows indicate relationships inferred from the graph.

Consider a piece of empirical medical knowledge: there is a hyperechoic area in patient A’s BUS images. We format this knowledge in the form of a triple:

(1) $$Knowledge=\left(patientA,\;echogenicity,\;hyperechoic\right)$$

where patientA denotes patient A, hyperechoic denotes there is a hyperechoic area in patient A’s BUS images, and echogenicity denotes that triple represents the relation about echogenicity. Then, we can abstract the representation of knowledge in the form of a triple from Equation (1):

(2) $$K=\left(h,\;r,\;t\right)$$

where h is called the head entity, r is called the relation, and t is called the tail entity. When a certain number of knowledge triples are collected, the knowledge graph can be constructed. A common practice in the medical field is to make all patients the head entities, make the different dimensional representations as the relations, and make the value corresponding to the representation the tail entity, which is as in Equation (1). When n pieces of knowledge are collected and aggregated into a knowledge set:

(3) $$S=\left\{K|K_{i}=\left(h_{i},\;r_{i},\;t_{i}\right),\;i=1,\;2,\;\cdots ,\;n\right\}$$

where $K_{i}$ is the ${ith}^{}$ piece of knowledge, $h_{i}$ is the ${ith}^{}$ head entity, $r_{i}$ is the ${ith}^{}$ relation, and $t_{i}$ is the ${ith}^{}$ tail entity. Usually, we classify both head and tail entities as entities, so the above set S can be expressed as a graph set G consisting of a set E of entities and a set R of relations:

(4) $$G=\left\{\left(E,R\right)|E=\left(h_{1},h_{2},\cdots ,h_{p},t_{1},t_{2},\cdots ,t_{q}\right),R=\left(r_{1},r_{2},\cdots ,r_{m}\right),p\in N^{*},q\in N^{*},m\in N^{*}\right\}$$

where p denotes the number of head entities, denotes the number of tail entities, and denotes the relations. So far, we can express the knowledge as a graph, i.e., use nodes to represent entities and edges to represent relationships to describe the knowledge graph figuratively. In this way, we can construct a simple knowledge graph of medical ultrasound image features using BI-RADS characteristics, as shown in Figure 2.

For the BUS image diagnosis, the task of the knowledge graph is to predict the connection between the unknown patient entity and pathology entities, which is called the knowledge graph completion.

### 2.5. Inference Based on TuckER

As mentioned above, in order to use medical knowledge for aiding diagnosis, we can predict the missing links by constructing a medical knowledge map and making complements to infer the diagnosis of the case. An algorithm based on tensor space embedding is a common choice. Such algorithms tensor entities and relations first, map them into a space of specified dimensions, and determine whether there are hidden links between the target entities by calculating and optimizing the spatial tensor distance. TuckER is a knowledge graph completion method based on embedding, proposed by Ivana et al. in 2019 [27]. They reconsidered the concept of Tucker tensor decomposition [30] and developed the TuckER model to decompose knowledge embeddings into three parts of tensors:

(5) $$\phi \left(h,r,t\right)=W\times_{1}h\times_{2}r\times_{3}t$$

where $\phi \left(h,r,t\right)$ denotes a piece of knowledge indicating there is a relation r from the head entity h to the tail entity t. After being mapped to the tensor space, h, r, and t represent h, r, and t, respectively. As a result, W becomes the weight tensor needed to be calculated to make the equation hold and is called the core tensor. Bernoulli negative log-likelihood loss function [31] is employed to train the model:

(6) $$L=-\frac{1}{n_{e}}\underset{i=1}{\overset{n_{e}}{\sum }}\left(y^{\left(i\right)}\log\left(p^{\left(i\right)}\right)+\left(1-y^{\left(i\right)}\right)\log\left(1-p^{\left(i\right)}\right)\right)$$

where $p^{\left(i\right)}$ is the predicted probabilities of the ${ith}^{}$ sample, and $y^{\left(i\right)}$ is the ground truth of the ${ith}^{}$ sample.

### 2.6. Tensor Fusion-Based Correction

In order to leverage the experienced knowledge of senior radiologists, we design a tensor distance-based correction mechanism. For a certain BUS image, a senior radiologist may give a more accurate diagnosis report than a junior one. Therefore, we used the BI-RADS score from senior radiologists as the gold standard to compare the BI-RADS score from junior radiologists for the same BUS images. In the tensor space, we can use the core tensor of the knowledge training of the senior radiologists to correct the core tensor of the knowledge training of the junior radiologists. Hence, we fused the BI-RADS scores of senior and junior radiologists in the training set to introduce a correction bias for improving the performance of inference.

### 2.7. Data Augmentation

Since tensor-decomposition-based models are sensitive to the positive and negative balance of samples, we used random undersampling and oversampling to balance the positive and negative samples. Unlike traditional structured data or image data, the head tensor of the knowledge tensor is real-named, i.e., the head entity corresponds to a particular case image, and, thus, simple replication of the data in oversampling is not useful for tensor space optimization. In view of this, we simulate more samples in oversampling by generating a fictitious new head tensor as the head entity of the duplicate knowledge.

### 2.8. Evaluation Metrics

Based on the definition and construction of the knowledge tensor above, assisted diagnosis can be defined as a multi-classification task. Therefore, we employ the common evaluation metrics based on the multi-classification, which are accuracy, precision, sensitivity, specificity, and F1 score. As the diagnosis target in this study is to infer whether the breast tumor is benign or malignant, which is a binary classification task, AUC is employed as the metric as well. For a binary classification task, there could be four basic statistical variables to describe the number of classification results, which are the true positive (*TP*), the true negative (*TN*), the false positive (*FP*), and the false negative (*FN*). The accuracy could be calculated by:

(7) $$Accuracy=\frac{TP}{TP+TN+FP+FN}$$

Correspondingly, the other indicators can be derived from the following equations:

(8) $$Precision=\frac{TP}{TP+FP}$$

(9) $$Recall=Sensitivity=\frac{TP}{TP+FN}$$

(10) $$Specificity=\frac{TN}{TN+FP}$$

(11) $$F1=\frac{2\times Precision\times Recall}{Precision+Recall}$$

*F*1 score and AUC usually provide a more objective and simultaneous response to the accuracy of positive and negative sample classification and are the metrics we are most interested in.

## 3. Results

### 3.1. Data Description

The dataset consists of 1219 cases with 3413 BUS images in total. Pathological examination was performed in all cases. There are 2667 malignant cases and 746 benign cases, respectively. A total of 1190 patients’ ages were recorded, with a mean value of 48.08, a minimum value of 13, and a maximum value of 87. For each BUS image, ten-dimension BI-RADS characteristics are labeled by radiologists, which are angular, calcification, distortion, indistinct, margin, micro-lobulation, orientation, posterior, shape, and speculation. The statistics of the dataset are shown in Table 1.

### 3.2. Knowledge Tensor-Aided Diagnosis Performance

**Figure 3:**
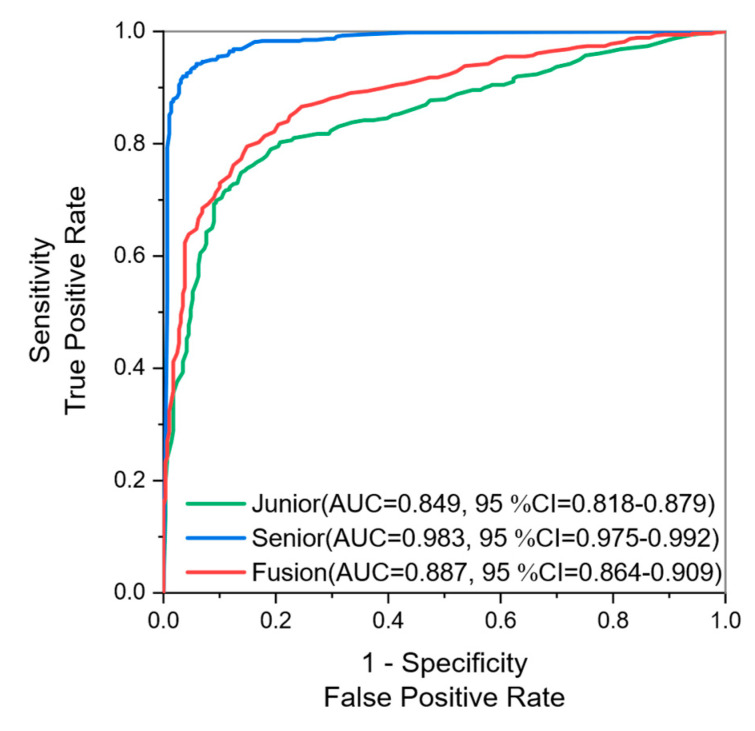
The ROC curve of test results with different knowledge tensors.

We conducted the experiments with the junior knowledge tensor, the senior knowledge tensor, and the fused knowledge tensor. As shown in Table 1, the proportion of benign and malignant cases is relatively disparate. Therefore, we used random undersampling to keep the ratio of benign to malignant samples at 1:1. This was because the TuckER model is calculated and optimized based on tensor space distance and, thus, is sensitive to the equilibrium of positive and negative samples, so we balanced the proportion of positive and negative samples. Through undersampling, the number of both benign and malignant images is set at 746 from 826 cases in total. We then utilize five-fold cross-validation to prevent overfitting and, thus, go on to better evaluate the accuracy of the auxiliary diagnosis. We find the optimal learning rate and the number of iterations through a grid search method. Test metrics of each method are shown in Table 2. The ROC curve is shown in Figure 3.

### 3.3. Comparison with Traditional Machine Learning Models

**Figure 4:**
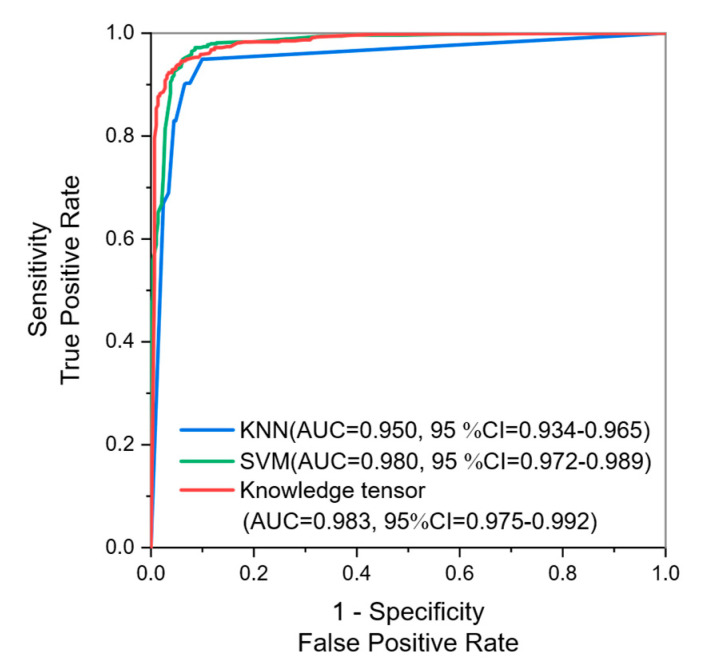
The ROC curve of test results with knowledge tensor and traditional methods.

Two traditional machine learning models, KNN and SVM, are employed to infer the diagnosis results with senior radiologists’ knowledge as well. The KNN model is pre-constructed using Scikit-learn [32,33]. The SVM model is implemented with LIBSVM [34]. Five-fold cross-validation is also conducted to prevent overfitting. Test metrics of each method are shown in Table 3. The ROC curve is shown in Figure 4.

## 4. Discussion

We provide a novel knowledge tensor-aided BUS image assistant inference framework in this study. To evaluate our framework, we conduct the experiments with two groups of annotations from senior and junior radiologists. Then, we will discuss the results, problems, and further studies.

Senior radiologists are experienced, so they can eliminate interference factors in the BUS images to obtain more accurate diagnosis results. In our experiments, the AUC of the diagnostic results achieved using senior radiologists’ annotation is as high as 0.983 (95% CI = 0.975–0.992). In contrast, the younger junior radiologists are inexperienced, with an AUC of 0.849 (95% CI = 0.823–0.876) for diagnosis on the same dataset. After knowledge tensor-based fusion, the AUC of junior radiologists is improved to 0.887 (95% CI = 0.864–0.909). From Table 3 and Figure 4, our proposed knowledge tensor-aided diagnosis algorithm could achieve better performance than KNN and SVM. The AUC of KNN is 0.950 (95% CI = 0.934–0.965), which is significantly less than one of the knowledge tensors with *p* < 0.001. The SVM model performs similarly to the knowledge tensor, thanks to the excellent few-shot data hyperplane classification ability of SVM, but the AUC is still inferior to the knowledge tensor, at 0.980 (95% CI = 0.972–0.989). The result indicates that the knowledge tensor could mine latent information in the data more efficiently. From Table 2, we can learn that the diagnostic sensitivity and specificity of senior radiologists are basically comparable, indicating that the ability of senior radiologists to judge benign and malignant tumors is basically the same. In contrast, the sensitivity of junior radiologists is significantly lower than specificity, and the judgment ability of junior radiologists is corrected after the tensor fusion.

The experimental results corroborate the theory that BUS is highly dependent on radiologists’ empirical knowledge. However, it takes a lot of time and financial resources to train a senior radiologist, and each senior radiologist develops from the experience of a junior radiologist. Therefore, we cannot expect to build a better and more standardized system of physician training to solve this problem for the time being. Young radiologists need to keep practicing in order to gain experience. Based on this, our proposed BI-RADS score correction mechanism based on knowledge tensor has a higher practical value. We can use the knowledge tensor decomposition to extract the experienced knowledge core of senior doctors and apply it to the clinical diagnosis of junior doctors so that the precious knowledge of senior radiologists is not limited to a single diagnosis but can be applied to other patient-treatment scenarios in a more generalized way.

However, there are still some problems that need to be solved through further research in the follow-up. First, our proposed framework can only use the high-quality BI-RADS scores of senior physicians to correct the diagnoses of junior physicians, which has some generalizability but lacks greater interpretability, and the BI-RADS scores are already recognized by physicians as a high-quality diagnostic aid with clinical implications [35]. The use of a tensor decomposition model to extract the core tensor somewhat reduces the interpretability of the BI-RADS score itself. Therefore, it would be important to give an interpretable tensor constraint space that maintains the interpretability of medical features while tensorizing them into a generalized model with generalization capability. Second, our proposed framework is limited to correcting the scoring results of junior doctors and cannot help them to improve their diagnostic experience and ability. It would be of better clinical value to construct a differentiated assessment system to help junior physicians identify overlooked features or lack of experience. Finally, our study was limited to the ten most significant dimensional characteristics of the BI-RADS and did not cover all the dimensions of the BI-RADS scoring system. Being able to construct models that cover all dimensions of the scoring system would give our framework better performance and stronger generalization performance.

## 5. Conclusions

In this study, we proposed a novel knowledge tensor-aided BUS image assistant inference framework. We utilized the medical semantic characteristics to construct knowledge graphs. To validate our framework, experiments were conducted on a BUS image dataset with BI-RADS annotations from senior and junior radiologists. The results indicated that our proposed framework could improve the inference performance of junior radiologists. Our framework can be applied to all medical-imaging-assisted diagnosis scenarios with diagnostic specifications and has a wide range of application scenarios.

## Figures and Tables

**Table 1:** Statistics of the BUS image dataset.

Statistic Item	Statistic Value
Number of cases	1219
With age recorded	1190 (97.62%)
Average ages with recording	48.08 (13–87)
Number of images	3413
Benign	746 (21.86%)
Malignant	2667 (78.14%)

**Table 2:** Performances of BUS diagnosis with junior knowledge tensor, senior knowledge tensor, and fused knowledge tensor. Data in parentheses are 95% confidence intervals (CIs). *p* values indicate the significance levels with 95% CI between our proposed framework and compared knowledge tensors.

Knowledge	Accuracy	Precision	Sensitivity	Specificity	F1 Score	AUC (95% CI)	*p* Value
Junior radiologist	0.791	0.893	0.768	0.832	0.826	0.849 (0.823–0.876)	<0.001
Senior radiologist	0.944	0.967	0.946	0.942	0.957	0.983 (0.975–0.992)	<0.001
Tensor-fused	0.809	0.909	0.783	0.856	0.841	0.887 (0.864–0.909)	-

**Table 3:** Performances of BUS diagnosis with senior knowledge. Data in parentheses are 95% CI. *p* values indicate the significance levels with 95% CI between knowledge tensor and traditional machine learning models.

Methods	Accuracy	Precision	Sensitivity	Specificity	F1 Score	AUC (95% CI)	*p* Value
KNN	0.874	0.972	0.830	0.955	0.895	0.950 (0.934–0.965)	<0.001
SVM	0.946	0.957	0.959	0.921	0.958	0.980 (0.972–0.989)	0.428
Knowledge tensor	0.944	0.967	0.946	0.942	0.957	0.983 (0.975–0.992)	-

## Data Availability

Our data are available at http://wisemed.cn.
